# Impingement by a polymyomatous uterus: a rare cause of large bowel obstruction

**DOI:** 10.1093/jscr/rjab182

**Published:** 2021-05-10

**Authors:** Lalita M Andersen, Christopher Y S Bong, Matthew J Burstow, Peter J Yuide

**Affiliations:** Department of Surgery, Logan Hospital, Meadowbrook, Australia

## Abstract

A primiparous female developed acute large bowel obstruction Day 1 post lower segment Caesarean section. Initially presumed to be post-operative ileus, increasing abdominal pain and distension over the next 24 h prompted a surgical consult. Computed tomography imaging demonstrated an abrupt transition point of the large bowel behind a polymyomatous uterus. Although this case resolved with vigorous patient mobilization, literature review reveals previous cases resolving only after intraoperative mobilization of the uterus. It is distinct from ileus as bowel sounds are present, onset is abrupt, progression is rapid and mobilization of the uterus causes immediate resolution. This condition is likely to be more common than the literature would suggest, its scarcity partially due to the reluctance to image young females especially during pregnancy.

## INTRODUCTION

Large bowel obstructions, while not as common as small bowel obstructions, still account for up to 4% of surgical admissions [1]. Of the known causes, over 60% are neoplastic, such as colorectal cancer, peritoneal carcinomatosis and local invasions from other malignancies [2]. Non-neoplastic causes include diverticulitis and volvulus, together accounting for 30% of causes [2]. The remaining causes that are frequently listed include intussusception, enteroliths, faecal impaction and endometriosis [1]. This case report presents a rare and controversial cause of large bowel obstruction—the uterus itself.

## CASE REPORT

A 35-year-old female was referred to the surgical team with 24 hours of increasing abdominal pain.

She had undergone emergency lower segment Caesarean section (LSCS) the day prior for preterm premature rupture of membranes. Eight hours post-operatively, the patient developed cramping abdominal pain with increasing abdominal distension, nausea and vomiting. At time of review, she had not passed flatus for 24 h and had not defecated for 4 days. She was primiparous, had no other known medical conditions and no previous surgeries.

**Figure 1:**
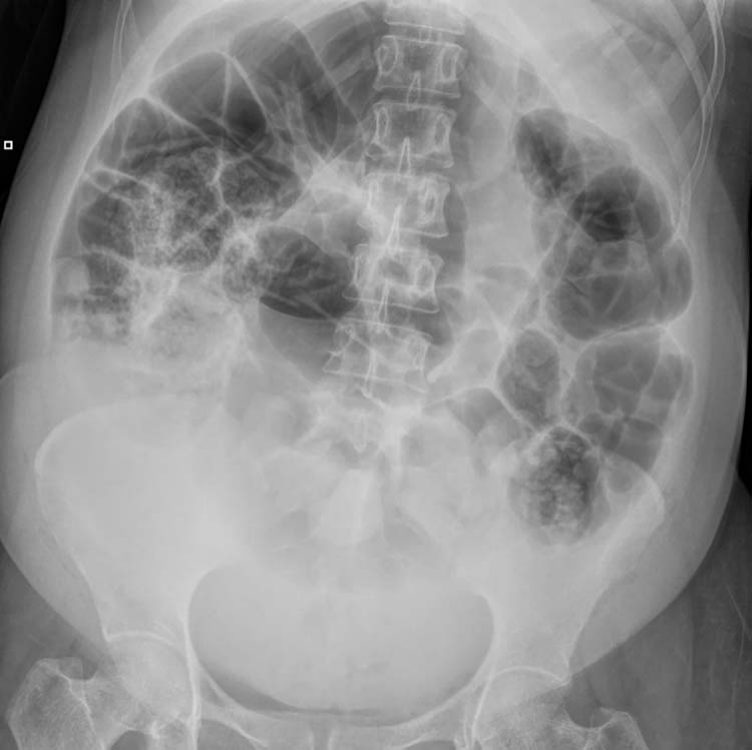
X-ray Abdomen, 24 h after commencement of symptoms. Widespread dilatation of large and small intestines stopping at the level of the pelvic brim. Moderate faecal loading in the caecum and descending colon.

On examination, she was in marked discomfort with a grossly distended abdomen. Despite generalized voluntary guarding, her abdomen was soft, with no focal peritonism. Bowel sounds were frequent and high-pitched, and percussion was tympanic throughout the upper abdomen. Abdominal X-ray by the treating Obstetrics team noted dilatation of the entire bowel above the pelvic brim, as shown in Fig. 1. Blood results were unremarkable, with Hb 113, WCC 17.9, CRP 64, K 4.1, Mg 0.81, and normal renal and liver function. A computed tomography (CT) abdomen and pelvis was organized to rule out mechanical obstruction.

**Figure 2:**
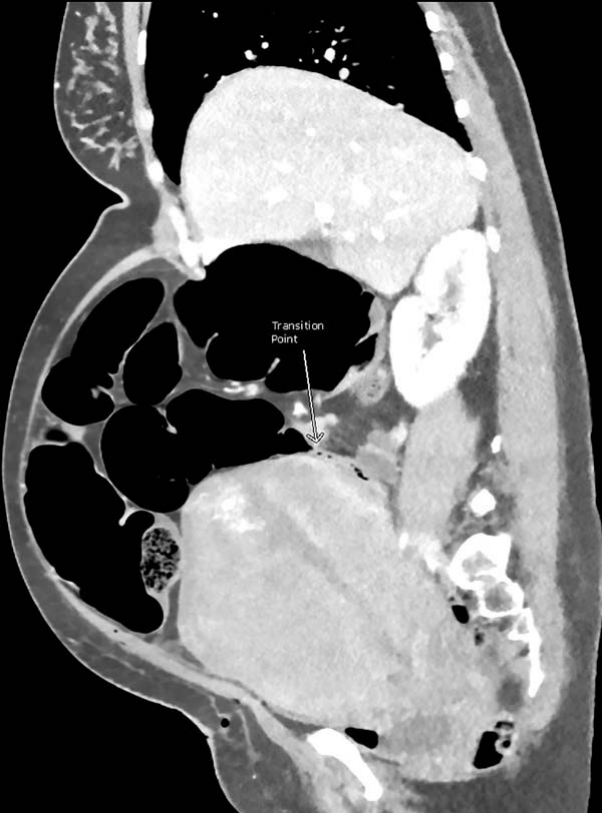
CT abdomen and pelvis with venous phase contrast, sagittal view. Abrupt collapse of the gas-filled sigmoid colon is shown posterior to the enlarged uterus. Subcutaneous gas in the anterior abdominal wall is from recent surgery.

**Figure 3:**
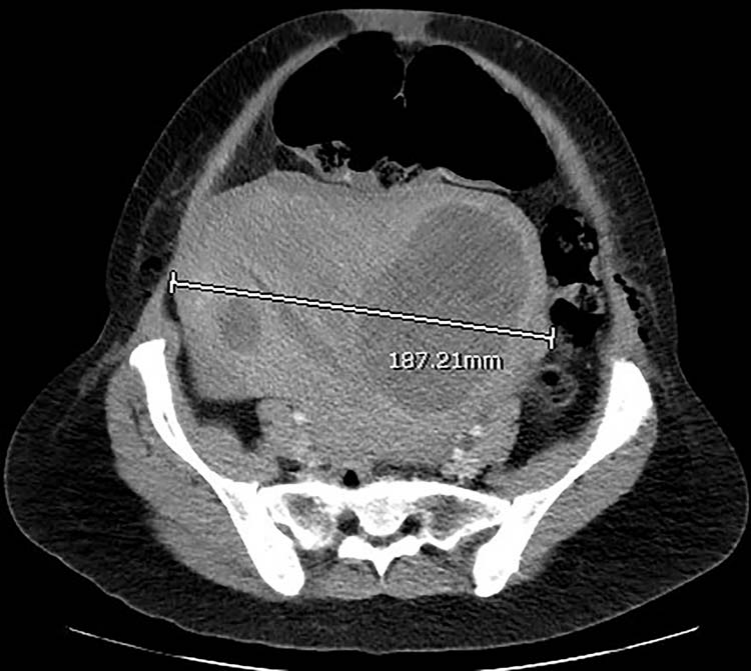
CT abdomen and pelvis with venous phase contrast, axial view. Polymyomatous uterus at its maximal width of 187.21 mm, 2 days postpartum. Note the larger fibroid on the right, reported to show necrotic changes. Dilated bowel loops visible anterior to the uterus.

CT images suggested complete obstruction of the sigmoid colon secondary to impingement by polymyomatous uterus (see Fig. 2). The transition point was directly posterior to a large fibroid which appeared necrotic, shown in Fig. 3. Although the pathology was arguably mechanical, it was decided to trial conservative management, consisting of NGT insertion, enemas, laxatives, electrolyte optimization, cessation of constipating medication and frequent mobilization. Within 24 h, the patient began to pass flatus. Her symptoms resolved completely after 72 h and she was discharged with gynaecology follow-up.

## DISCUSSION

Complete bowel obstruction caused by the uterus alone is an elusive condition that has not been proven on imaging until this day. The concept was introduced to English readers through reviews of German and French literature by Blair in 1932, and Eliason and Erb in 1937 [3, 4]. They suggested the controversial belief that the uterus on its own can cause intestinal obstruction, by collecting 235 French or German reports of bowel obstruction in pregnancy, 48 of which were presumed due to compression by the uterus alone [3, 4]. In the French and German studies, the diagnosis of obstruction from the uterus was made either intraoperatively or at autopsy [3, 4]. Since then, between 1920 and 2020, 12 case reports in English academic literature have described this impingement of the sigmoid colon between uterus and pelvic brim, as summarized in Table 1 [3–10].

**Table 1:** Case reports of bowel obstruction secondary to uterine impingement, 1920–2020

Author/year	Cause of obstruction	Definitive management
Blair/1932 [3]	7 months gravid uterus.	Enemas—failed. Laparotomy: manual straightening of sigmoid colon, insertion of rectal tube. Vaginal delivery 2 days post-operatively.
Eliason/1937 [4]	25 weeks gravid uterus.	Caecostomy—failed. Laparotomy: premature delivery and abortion of fetus.
Eliason/1937	4 months gravid uterus.	Soapsud enemas in knee–chest position.
Eliason/1937	6 months gravid uterus.	Enemas in right lateral position with bed tilted head-down.
Jackson/1920 [5]	Bicornuate uterus with 2 month old fetus in abnormal horn.	Enemas—failed. Laparotomy: Delivery and abortion of fetus from abnormal horn.
Ghosal/1931 [6]	Retroverted uterus, menstruating.	Rectal tube, Hobb’s intra-uterine glycerine, prone position for 3 weeks.
Ghosal/1931	Retroverted uterus, 3–4 weeks postpartum.	Rectal tube, Hobb’s intra-uterine glycerine, prone position for 3 weeks.
Ghosal/1931	Retroverted uterus, 3 months gravid.	Rectal tube, abortion, pessary.
Dan/1988 [8]	30 weeks gravid uterus (triplets), prolonged bed rest, tocolytics.	Right lateral position—failed. Laparotomy: premature delivery, rectal tube.
Gonzalez-Mesa/2013 [9]	33 weeks gravid uterus, dextroflexed.	Postural changes, rectal tube, spasmolytics, NGT–failed. Laparotomy: premature delivery.
Adamou/2018 [7]	Polymyomatous uterus. Concurrent ruptured right tubal ectopic pregnancy.	Laparotomy: total hysterectomy.
Li/2018 [10]	Normal uterus, 2 days post LSCS.	Right lateral decubitus position overnight.
Andersen/2021 (current case)	Multifibroid uterus, 1 day post-LSCS.	Mobilization, NGT and enemas.

In order to cause a mechanical obstruction, the uteri in these cases were either physiologically or pathologically enlarged. A bicornuate uterus with an 8 week fetus in one of its horns caused a large bowel obstruction until the fetus was surgically aborted [5]. Ghosal reported three cases of obstruction by retroverted uteri, enlarged either due to pregnancy, swelling in postpartum period or even menstruation [6]. Ghosal’s cases all resolved with rectal tubes and sleeping in the prone position for over 3 weeks [6]. A polymyomatous uterus such as in our case has been reported previously, though this was complicated by a ruptured tubal ectopic pregnancy, which may have contributed to the obstruction [7]. The remainder of the obstructions were in normal but pregnant uteri, ranging from 16 weeks to 35 weeks gravidity [3, 4, 8, 9]. Two of the pregnancies were complicated, one with a detroflexed uterus, another holding a triple pregnancy [8, 9]. A normal uterus 2 days post LSCS was also shown to cause bowel obstruction. Though the obstruction was not complete—the patient was still passing flatus—this case is worthy of mention as the only case to date which demonstrates this uterine impingement on CT imaging [10].

Many authors have emphasized the importance of imaging to define the pathology and direct appropriate management [4, 10, 11]. Abdominal plain films were suggestive of uterine impingement when demonstrating dilatation of the entire bowel above the pelvis [7, 8]. Barium enemas would show an abrupt obstruction at the rectosigmoid junction [4]. Magnetic resonance imaging has not yet shown uterine impingement of the colon, however, a case of small bowel obstruction by a gravid uterus has been reported [11]. CT imaging was uncommon in this cohort as many of the patients were pregnant, with only one partial obstruction demonstrated in the postpartum period [10]. This case is the first to demonstrate complete obstruction of the colon from uterine impingement on CT imaging.

There is no consensus on the management of this condition, however specific successful methods include rectal tubes, enemas in knee–chest position, or putting the patient in right lateral or prone positions [4, 6, 10]. Half the cases reported progressed to laparotomy, either based on a presumed surgical diagnosis, or due to failed conservative management [3–5, 7–9]. Intraoperatively, the large bowel was found to be dilatated up to the point where the sigmoid colon crossed the pelvic brim, resolving the moment the uterus was mobilized [3–5, 7–9]. Although these laparotomies did not find surgical pathology, some patients still underwent an unplanned Caesarean section, abortion or hysterectomy as a result [4, 5, 9]. With increased awareness of this condition, and early diagnosis on imaging, we hope that future cases will be able to be avoid these unwanted outcomes.

## References

[1] Ramanathan S , Ojili V, Vassa R, Nagar A. Large bowel obstruction in the emergency department: imaging spectrum of common and uncommon causes. J Clin Imaging Sci 2017;7:15. 28480123 10.4103/jcis.JCIS_6_17 PMC5404618

[2] Catena F , De Simone B, Coccolini F, Di Saverio S, Sartelli M, Ansaloni L. Bowel obstruction: a narrative review for all physicians. World J Emerg Surg 2019;14:20. 31168315 10.1186/s13017-019-0240-7 PMC6489175

[3] Blair M . Intestinal obstruction caused by normal pregnancy. Can Med Assoc J 1932;26:426–9. 20318679 PMC402288

[4] Eliason EL , Erb WH. Intestinal obstruction complicating pregnancy. Surgery 1937;1:65–73.

[5] Jackson CE . Acute intestinal obstruction due to pregnancy in a bicornuate uterus. Br Med J 1920;1:185. 20769785 10.1136/bmj.1.3084.185 PMC2337177

[6] Ghosal JN . Intestinal obstruction caused by retroverted uterus. Ind Med Gaz 1931;66:509. PMC5185782 29010032

[7] Adamou H , Amadou Magagi I, Oumarou Garba S, Habou O. Acute intestinal obstruction due to extrinsic compression by previa myoma and ectopic pregnancy: a case report. J Med Case Rep 2018;12:10. 29335010 10.1186/s13256-017-1540-8 PMC5769554

[8] Dan U , Rabinovici J, Koller M, Barkai G, Mashiach S. Iatrogenic mechanical ileus due to over-distended uterus. Gynecol Obstet Invest 1988;25:143–4. 3371763 10.1159/000293762

[9] González-Mesa E , Narbona I, Cohen I, Villegas E, Cuenca C. Uterine rotation: a cause of intestinal obstruction. Case Rep Obstet Gynecol 2013;2013:759250. 23781360 10.1155/2013/759250 PMC3676995

[10] Li R , Dooreemeah D, Cichowitz A. The post partum uterus – a rare cause of mechanical large bowel obstruction. Austin J Surg 2018;5:1122.

[11] Daimon A , Terai Y, Nagayasu Y, Okamoto A, Sano T, Suzuki Y, et al. A case of intestinal obstruction in pregnancy diagnosed by MRI and treated by intravenous hyperalimentation. Case Rep Obstet Gynecol 2016;2016:8704035. 27999695 10.1155/2016/8704035 PMC5143715

